# First Evidence for a Massive Extinction Event Affecting Bees Close to the K-T Boundary

**DOI:** 10.1371/journal.pone.0076683

**Published:** 2013-10-23

**Authors:** Sandra M. Rehan, Remko Leys, Michael P. Schwarz

**Affiliations:** 1 Department of Biological Sciences, University of New Hampshire, Durham, New Hampshire, United States of America; 2 School of Biological Sciences, Flinders University of South Australia, Adelaide, South Australia, Australia; 3 Evolutionary Biology Unit, South Australia Museum, Adelaide, South Australia, Australia; 4 School of Earth and Environmental Sciences, University of Adelaide, Adelaide, South Australia, Australia; Onderstepoort Veterinary Institute, South Africa

## Abstract

Bees and eudicot plants both arose in the mid-late Cretaceous, and their co-evolutionary relationships have often been assumed as an important element in the rise of flowering plants. Given the near-complete dependence of bees on eudicots we would expect that major extinction events affecting the latter would have also impacted bees. However, given the very patchy distribution of bees in the fossil record, identifying any such extinctions using fossils is very problematic. Here we use molecular phylogenetic analyses to show that one bee group, the Xylocopinae, originated in the mid-Cretaceous, coinciding with the early radiation of the eudicots. Lineage through time analyses for this bee subfamily show very early diversification, followed by a long period of seemingly no radiation and then followed by rapid diversification in each of the four constituent tribes. These patterns are consistent with both a long-fuse model of radiation and a massive extinction event close to the K-T boundary. We argue that massive extinction is much more plausible than a long fuse, given the historical biogeography of these bees and the diversity of ecological niches that they occupy. Our results suggest that events near the K-T boundary would have disrupted many plant-bee relationships, with major consequences for the subsequent evolution of eudicots and their pollinators.

## Introduction

Recent molecular phylogenetic studies of bees have greatly changed our understanding of how this key pollinator group has evolved [1]. Major advances have been made in understanding the timing of their origin [2], estimating the number and timings of transitions to sociality [3]–[7], the effects of nest-construction methods on diversification patterns [8], the likely ancestral state for breadth of floral resource use [9], phylogeographic history [10]–[13], and temporal patterns of diversification [11], [12], [14], [15].

Because bees are such important pollinators of eudicots, their phylogeography and patterns of diversification over time are important for understanding how modern terrestrial communities have evolved. Cardinal and Danforth [2] have recently shown that bees originated in the mid-late Cretaceous, at a time when the eudicots were also diversifying. Cardinal and Danforth [5] showed that most subfamilies and tribes in the long-tongued bee family Apidae arose in the Late Cretaceous or later, but they did not explore rates of diversification. Almeida et al. [10] examined diversification rates in the short-tongued bee family Colletidae, extending back to the Late Cretaceous, about 70 Mya. They found evidence for late-stage Gondwanan interchanges involving Australia, Antarctica and South America, and patterns of cladogenesis indicated a period of accelerated diversification in the last 25–30 Mya, corresponding to increasing aridification in the Southern Hemisphere [16]–[19], where colletids are particularly diverse [9]. However, they did not find evidence of an extinction event corresponding to the K-T boundary.

Studies of angiosperms have suggested a massive extinction event [20]–[22] that is close to the K-T boundary, where a bolide impact has been implicated in the extinction of the non-avian dinosaurs [23]. However, major episodes of global climate instability began about 1 million years before the K-T boundary [24], including a drop of 6–8°C approximately 100 Ky before the K-T [25] and dramatic changes in sea levels span the K-T [26]. Consequently, the clustering of multiple major changes either side of the K-T boundary may be implicated in extinction events close to that time, rather than just a single event. Regardless of how different factors may have combined to alter ecosystems, there is evidence that extinction events close to K-T disrupted a large number of insect-plant relationships [22], but see [27], [28] for evidence that many insect groups did not seem to suffer large extinctions at this time.

Given the close relationship between eudicots and bees, one might expect that any extinction events affecting eudicots would also impact on bees and vice versa. Rapid and simultaneous extinctions in both bees and their host plants would have affected plant-pollinator dynamics in ways that could shape subsequent ecosystems in very important ways [29]. For example, extinction of plant-specialist (oligolectic) bees would have impacted strongly on their dependent hosts, whereas loss of generalist (polylectic) bee pollinators would have had more diffuse effects [30], [31]. In both cases, large reductions in the numbers of both eudicots and their pollinators would have introduced a strong stochastic element to how ecosystems subsequently reassembled.

Exploring how bee diversification rates may have varied since the Cretaceous requires a bee group whose history extends back to that time, but it also requires a group where taxon sampling is sufficiently dense to recover diversification patterns. Our extended research group has used molecular phylogenetics to explore social evolution in the long-tongued bee family Xylocopinae [4], [6], [32], [33] and as a result we have obtained DNA sequence data for a large number of species in each of the four constituent tribes (summarized in Table S1). Our resulting samples cover most of the described genera and subgenera in this subfamily, and the resulting sample of 229 species is the most comprehensive sample of sequences for any bee subfamily that we are aware of. The crown age of the Xylocopinae has been dated at ca. 90 Mya by Cardinal et al. [34] and ca. 85 Mya by Cardinal and Danforth [2], during the mid Cretaceous. Here we use our extensive sequence data from Xylocopinae species, covering all four extant tribes, to explore patterns of bee cladogenesis over a time period that crosses the K-T boundary.

## Methods

### Included taxa

Our ingroup included all Xylocopinae species for which we had sequence data, totaling 229 species. These species are listed in Table S1 along with NCBI accession numbers for the gene fragments used in our analyses. Our taxa covered all species of Manueliini, and inclusion of species in the three other tribes was based on availability of sequence data and the desirability of representing as wide a range as possible of the major intra-tribal clades identified by previous studies [11], [12], [32].

Our outgroup comprised five species from the corbiculate long-tongued bees, *Apis mellifera, Bombus terrestris*, an undescribed Malagasy *Liotrigona* species, and two euglossine species. These species provide representation of all four corbiculate tribes, Apini, Bombini, Meliponini and Euglossini respectively. Previous analyses firmly indicate that these four tribes comprise a monophyletic clade that does not include the Xylocopinae [5].

### Gene fragments

Four gene fragments were used in our phylogenetic analyses: two mitochondrial genes, cytochrome oxidase subunit 1 (COI –1279 base pairs) and cytochrome b (cytb –428 base pairs), and two nuclear genes, the F1 and F2 copies of Elongation Factor-1α (EF-1α), with 460 and 772 base pairs respectively. However, not all fragments were available for all species. Complete fragments were obtained for 83/229 or 36% of all species (Table S1). DNA extraction, PCR amplification and sequencing were performed as described in Leys et al. [32], Schwarz et al. [33] and Rehan et al. [12]. The intron region of the F2 copy of EF-1α was largely unalignable and was not included in the analyses.

### Phylogenetic analyses

We used two methods to develop chronograms of our taxa. Firstly we used a Bayesian Monte Carlo Markov Chain (MCMC) approach, using a lognormal relaxed clock model implemented in BEAST version 1.6.2 [35]. We used nine partitions for this, comprising 1^st^, 2^nd^ and 3^rd^ codon positions for the combined mitochondrial genes, and 1^st^, 2^nd^ and 3^rd^ codon position for each of the two nuclear genes. On the basis of earlier analyses using both hierarchical LnL and AIC analyses in ModelTest 3.06, we used a GTR +I+Γ model for each partition, with all parameters subsequently estimated in BEAST. We fitted a GTR +I+Γ model to each partition and substitution parameters were unlinked. We used 50 million iterations in our MCMC analyses, sampling every 10,000^th^ iteration to reduce autocorrelation in parameter estimates. We examined parameters and model LnL values across sampled iterations using Tracer 1.5 to determine an appropriate burnin [36].

To examine whether topologies and chronograms from BEAST were robust to different assumptions, we also undertook MCMC Bayesian analyses using MrBayes 3.2.0 [36]. For these analyses COI and cytb were combined, as in our BEAST analysis, and then the combined mitochondrial and each of the nuclear genes were separately partitioned into 1^st^ +2^nd^, and 3^rd^ codon positions, giving a total of six partitions. We used default MrBayes priors (37), with a GTR +I+Γ model for each partition, and partitions were unlinked for all substitution model parameters. We ran one cold and three heated chains for 35 million generations, sampling every 10,000th iteration. Stationarity in model parameters was assessed as for the BEAST analysis, using trace plots of parameters in Tracer 1.5 [36].

Whilst our BEAST analyses produce ultrameric trees that can be interpreted as chronograms, phylogenies from the MrBayes analyses required transformation of phylograms to produce chronograms. We transformed the MrBayes consensus phylogram and the last 200 postburnin phylograms (covering the last 2 million iterations) into chronograms using Sanderson's penalised likelihood (PL) transformation with a cross validation procedure to choose a smoothing value, implemented in r8s version 1.71 [38]. Exploratory analyses indicated that chronogram branch lengths were sensitive to smoothing values and we used multiple cross validation runs to locate an appropriate value across a range spanning zero to 1000. There are no reliable fossils within the extant Xylocopinae tribes that can be used as calibration points, but there is an extinct tribe Boreallodapini from Baltic amber, dated at 45.1 Mya, which is sister tribe to the Allodapini [39], which allows us to set a minimum age for divergence between Allodapini and Ceratinini. Preliminary runs in BEAST always yielded trees where the divergence between Allodapini and Ceratinini was much older than the minimum 45 Mya date that this fossil requires, so in the final three analyses this calibration point was not enforced. For both the BEAST and r8s analyses we set the root node, comprising the most recent common ancestor of the Xylocopinae and the corbiculate apids, at 107 Mya, which is the estimated age of this node in Cardinal and Danforth's [5] extensive molecular phylogenetic study of the Apidae. However, we also explored the effect of varying this node age between 90 and 120 My, which respectively approximate the lower 95% HPD for the node age of the MRCA of Xylocopinae and the corbiculates in Cardinal and Danforth [2] and the upper 95% HPD for the MRCA of these two groups in Cardinal et al. [34]. We note, however, that an MRCA age of 90 Mya might be unrealistically recent, given that some fossil Clusiaceae dated at ca. 90 Mya contain a suite of floral traits that strongly suggest pollination by the corbiculate tribes Euglossini and Meliponini [40]. Divergence between the corbiculates and Xylocopinae would therefore require corbiculate pollination modes to have both arisen and become coadapted with Clusiaceae floral morphology at about the same time that the corbiculate clade diverged from the lineage leading to the Xylocopinae.

### Diversification rates

We used a combination of lineage-through-time (LTT) plots and diversification models to explore diversification rates for Xylocopinae in detail. We used the mltt.plot command in APE [41] in the R statistical environment to generate LTT plots for both the maximum credibility chronogram, as well as 200 post-burnin chronograms used to examine credibility envelopes for LTT plot variations. The same approach was taken for the consensus phylogram and 200 post-burnin phylograms from our MrBayes analysis, all subjected to penalized likelihood transformation, as described above.

Our LTT analyses involve two kinds of uncertainty that are potentially important for inferring patterns in temporal diversification; firstly there is uncertainty in the estimated root node of the Xylocopinae and, secondly, there is uncertainty in how well a maximum credibility or consensus phylogram might represent the actual pattern of diversification given any particular root-node age and given the existence of phylogenetic uncertainty (see below) in some parts of the tree. One approach for addressing these uncertainties would be to set a root-node prior that proposes a most-likely age along with an age distribution from which the node age can be sampled; for example a mean age of 107 Mya with a normally-distributed standard deviation of 10 My. The problem with such an approach is that when examining multiple post-burnin LTT plots to develop a credibility envelope of diversification patterns, any variation in the lag-time between the root node and subsequent LTT curves will be obscured by variation in the root age itself. In order to partition these two sources of variation we used the following procedure. For the BEAST analyses we used the maximum credibility chronogram to infer a root node age for the Xylocopinae and then set this value as root age for the 200 post-burnin plots for developing credibility envelopes. For the MrBayes/r8s analyses we adopted the same approach, but scaled tree height for the post-burnin samples according to the value determined by analysis of the consensus phylogram. This approach allowed us to separately explore the shape of LTT curves and the effects of varying root node age on inferred crown ages for the various tribes.

### Simulations of a massive extinction event and long fuse models

Tree simulations have been used to explore whether anti-sigmoidal LTT plots may be due to massive extinction events (MEE) or a ‘long-fuse’ model (LFM) of diversification [42]. Stadler [43] provides a method for correctly conducting and sampling such tree simulations, but shows that such simulations may not allow LFM scenarios to be discriminated from MEEs. Despite these problems it is still important to explore the ability of these two kinds of models to predict observed patterns [44] and for our study it was important to examine how the timing of an extinction event is related to the apparent sudden increase in lineage accumulation after this event.

We used TreeSim version 1.7 [45] to compare LTT plots under a simulated MEE to a simulated LFM. Analyses were run three times to check for convergent model outcomes. For the MEE we set the extinction event at 65 Mya, and explored the effect of varying the proportion of lineages going extinct. For the LFM we assumed the same constant birth/death (λ/μ) ratios prior to 90 Mya and after 65 Mya, but varied this ratio for the period of 90–65 Mya. Determining an appropriate birth/death ratio on the basis of our recovered chronograms from BEAST and MrBayes is problematic because we had incomplete taxon sampling. We estimated possible values by pruning our maximum credibility BEAST chronogram to produce separate chronograms for the tribes Xylocopini, Ceratinini and Allodapini and then used the birth-death command in APE applied to the maximum credibility tree for each tribe. We did not include Manueliini in these analyses because it comprises only three extant species, and therefore only two internal nodes, which is insufficient to meaningfully estimate birth/death ratios. These analyses indicated a very wide range of possible λ/μ ratios, with upper 95% confidence limits ranging from approximately 2.5 to 7.

Because of the difficulty in choosing an appropriate λ/μ ratio based on our empirical chronogram, we visually explored simulated LTT plots under a wide range of λ/μ ratios ≤7 and compared these to our observed LTT plots. There are approximately 1000 described species of Xylocopinae [46], but this figure is only rough, given the likelihood of both undescribed species (e.g. [47]) and synonymies. In our simulations we therefore set the total number of extant species at 1000 and pruned the sampled number of species back to 200, using the TreeSim command sim.rateshift.taxa [45]. We found that a ratio of λ/μ = 2, with λ set at 0.2 and with an extinction rate of 92% produced an LTT plot that closely corresponded to our observed plots. We then used these values in the LFM simulations for times prior to 90 Mya and after 65 Mya, but varying the absolute values of λ and μ for the intermediate period.

## Results

**Figure 1:**
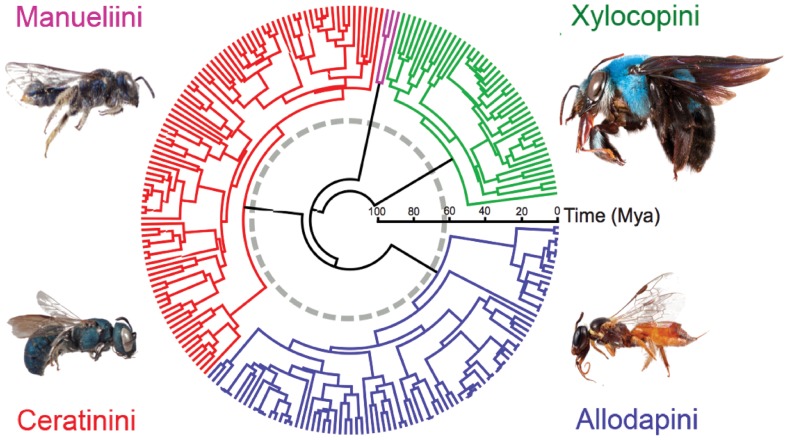
Chronogram from the BEAST uncorrelated log normal relaxed clock analysis. The outgroup, corbiculate apids, have been removed and the four Xylocopinae tribes are colour coded. The root node was set at 107*Ceratina unimaculata* (Ceratinini), *Allodapula rozeni* (Allodapini), *Manuelia gayi* (Manueliini) and *Xylocopa* sp. (Xylocopini).

The maximum credibility chronogram from our Bayesian BEAST analysis is summarized in Figure 1, where the four Xylocopinae tribes have color-coded branches and the K-T boundary and the late Eocene transition period are indicated by circles. This phylogeny with specimen nomina and posterior probability values for nodes is also given in Figure S1. The corresponding chronogram from penalized likelihood transformation of the MrBayes consensus phylogram is given in Figure S2, along with PP values for each node. Monophyly of all tribes is highly supported (PP = 1.0 for each tribal node) in both analyses, but many infra-tribal nodes had low support (PP<0.90).

Crown ages for all four xylocopine tribes based on the BEAST and MrBayes/r8s analyses are given in Table 1 and 95% credibility intervals for node ages for the BEAST analysis are indicated in Figure S3. Because the only reliable internal fossil calibration point for the Xylocopinae entailed a minimum divergence age of 45 Mya for the Allodapini and Ceratinini [33], [39] which is much younger than in any of our analyses, tribe crown ages are almost entirely determined by the value set for the root node, namely the divergence between lineages leading to the Xylocopinae and the corbiculate apines (Euglossini, Bombini, Meliponini and Apini). Figure 1 divergence dates are based on setting this node to 107 Mya which was the point-estimate derived by Cardinal et al. [34], but we also explored the effect of varying this value between 90 Mya and 120 Mya, which covers the range of 95% HPD limits for this MRCA in both Cardinal et al. [34] and Cardinal and Danforth [2].

**Table 1:** Age estimates of Xylocopinae root age and tribal crown ages obtained from an uncorrelated log normal relaxed clock model from BEAST as well a penalized likelihood transformation of a Bayesian phylogram from MrBayes (in parentheses).

	Root node:			
Crown ages:	90 My	100 My	107 My	120My
Xylocopinae	85.59	95.1	**101.8**	114.1
	(85.1)	(94.6)	**(101.2)**	(113.5)
Xylocopini	42.72	47.5	**50.8**	57.0
	(49.6)	(55.1)	**(58.9)**	(66.1)
Manueliini	33.92	37.7	**40.3**	45.2
	(45.1)	(50.1)	**(53.6)**	(60.1)
Ceratinini	48.41	53.8	**57.6**	64.5
	(52.0)	(57.8)	**(61.8)**	(69.3)
Allodapini	50.69	56.3	**60.3**	67.6
	(46.8)	(51.9)	**(55.6)**	(62.3)

The root node, connecting the corbiculate outgroup with the Xylocopinae, was set at four different values, ranging from 90 Mya to 120 Mya to explore the effects on internal node estimates. The root node age set to 107 Mya corresponds to the estimate for this node by Cardinal and Danforth [5].

**Figure 2:**
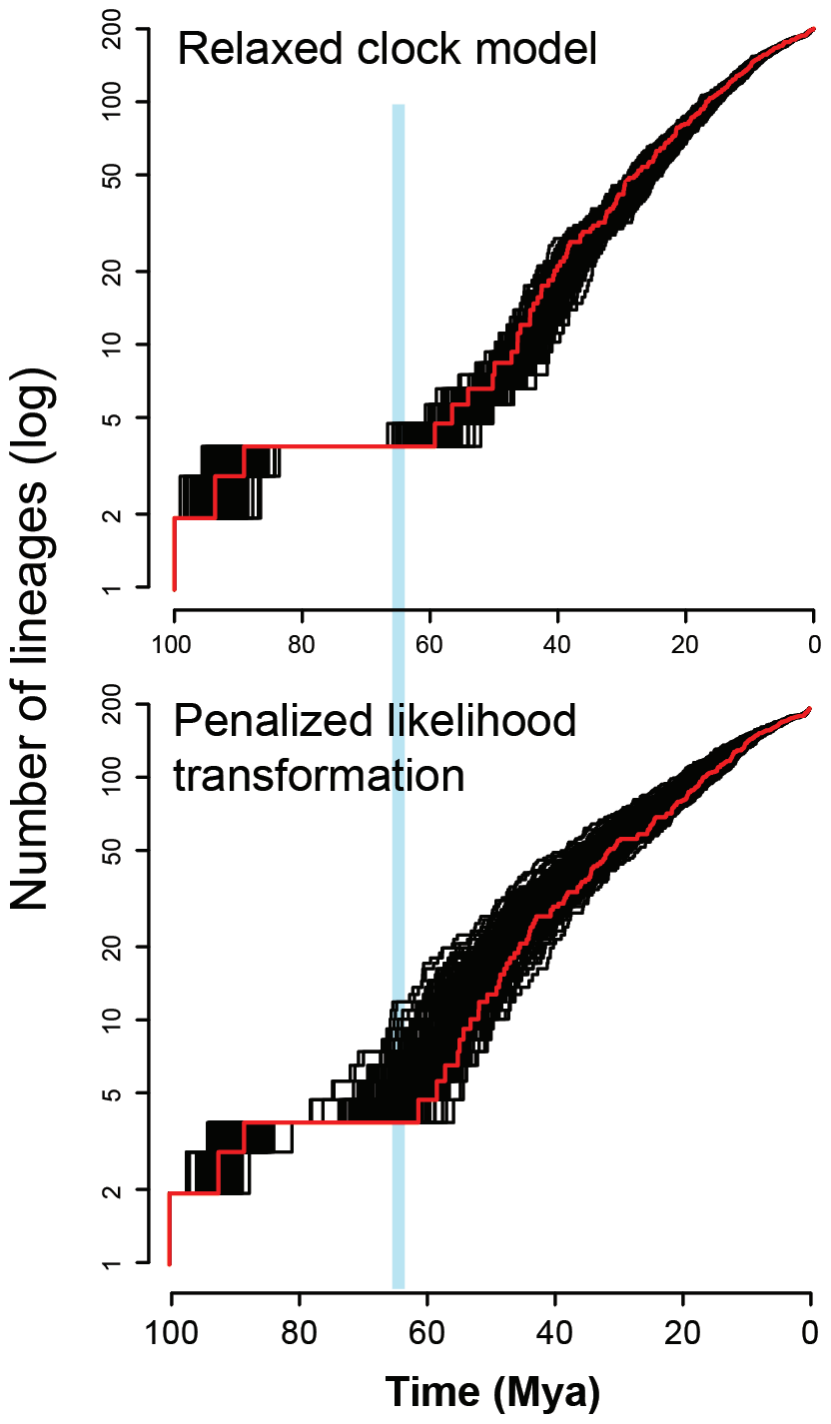
Log lineage through time (LTT) plots for Xylocopinae. Top panel gives results for an uncorrelated log normal relaxed clock (RC) model and the bottom panel gives results for a penalized likelihood (PL) transformation of MrBayes phylograms. The red lines indicate the maximum credibility tree for the RC analysis in the top panel and the PL-transformed consensus phylogram for the MrBayes/r8s analysis. Black lines indicate LTT plots for 200 post-burnin trees from each analysis. The blue line represents the K-T boundary.

The log lineage through time (LTT) plots of the maximum credibility chronogram of our BEAST analysis and the MrBayes/r8s chronogram, based on a root node age of 107 Mya, are given in Figure 2, along with credibility envelopes based on 200 post-burnin chronograms from each analysis. These plots show an anti-sigmoidal pattern, indicating a well-defined early episode of cladogenesis for the Xylocopinae starting about 100 Mya, followed by an apparent hiatus in radiations for the next ∼35–40 My, and subsequently followed by a sharp increase in cladogenesis centered on ∼3–5 My after the K-T boundary. Importantly, the credibility envelopes indicate that the anti-sigmoidal shape of this curve, and the times at which apparent radiation rates changed, are robust to the phylogenetic uncertainty present in some infra-tribal nodes.

**Figure 3:**
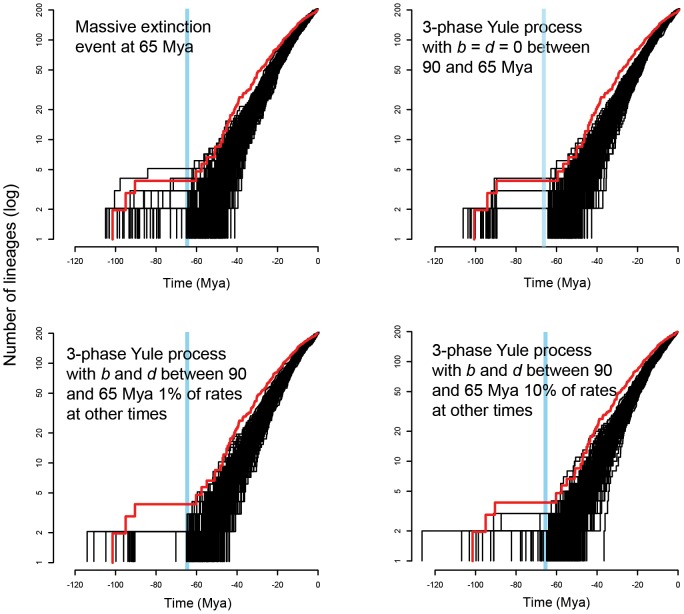
LTT plots for TreeSim simulations of a massive extinction event and three scenarios involving a 3-phase process where birth/death (λ/μ) values vary over time. Simulations specified 1000 actual extant taxa, pruned to 200 taxa to represent incomplete taxon sampling. (a) A massive extinction event at 65 Mya where λ = 0.2, μ = 0.1, and 92% of lineages go extinct at 65 Mya. (b) A 3-phase Yule process where λ = 0.2, μ = 0.1 prior to 65 Mya, λ = 0.0, μ = 0.0 between 90 and 65 Mya and where λ = 0.2, μ = 0.1 after 65 Mya. (c) A 3-phase Yule process where λ = 0.2, μ = 0.1 prior to 65 Mya, λ = 0.02, μ = 0.01 between 90 and 65 Mya and where λ = 0.2, μ = 0.1 after 65 Mya. (d) A 3-phase Yule process where λ = 0.2, μ = 0.1 prior to 65 Mya, λ = 0.002, μ = 0.001 between 90 and 65 Mya and where λ = 0.2, μ = 0.1 after 65 Mya. The red lines show the empirical LTT plots from Figure 2 based on the maximum credibility BEAST tree.

The anti-sigmoidal shape of our LTT plots could be interpreted as indicating either a ‘long-fuse’ model (LFM) of diversification, or a massive extinction event (MEE) [42] shortly preceding the apparent resumption of phylogenetic radiation after the LTT plateau [43], [44]. A long fuse model would involve a 3-phase Yule process where cladogenesis is initially high, is then followed by a period of very low speciation and extinction rates, and then followed by a second period of high rates of diversification. Our simulations (Figure 3) indicate that both LFM and MEE scenarios have the potential to explain our observed LTT patterns, and it has already been shown that simulation approaches may not, in themselves, be sufficient to discriminate between these alternatives [43].

Lastly, in Figure S4 we present LTT plots for the same 200 MEE simulations presented in Figure 3a, but with both extinct and extant lineages included. This figure provides some indication of the range in lineage numbers that could have been present immediately before any extinction event where 92% of lineages died out at 65 Mya, but where subsequent Yule processes lead to 1000 species in the present. This figure indicates a wide range in the likely number of lineages immediately prior to extinction, but most simulations contained more than 10 and less than 100 species.

## Discussion

Our analyses indicate two major features in the evolutionary history of the Xylocopinae. Firstly, the Middle Cretaceous crown age of this subfamily, leading to divergence of the four extant tribes, corresponds well in time with the major period of diversification of the eudicots [48]–[50]. Secondly, our LTT analyses show a very strong anti-sigmoidal curve, and the maximum credibility plots suggest that the actual or seeming resumption of high rates of cladogenesis after the apparent long-fuse period occurs at a time shortly after the K-T boundary. Therefore, our BEAST and MrBayes/r8s analyses both indicate very similar anti-sigmoidal patterns in apparent radiation of the Xylocopinae, both indicate that the mostly likely seeming resumption of high radiation rates occurred shortly after the K-T boundary, but credibility envelopes allow such an apparent resumption to lag behind the K-T event. Three important issues arise when interpreting these patterns: (i) the level of confidence in the root-node age; (ii) the extent of any lag-time between an extinction event, or change in birth/death rates, and the time when this shows up in LTT curves; and (iii) whether the anti-sigmoidal patterns are best explained via a long fuse model (LFM) or a massive extinction event (MEE). We now discuss these three issues.

The time axes in Figures 1 and 2 are based on setting the root node to 107 Mya from Cardinal et al. [12], whereas the more recent study by Cardinal and Danforth [2] suggests this node occurred at about 95 Mya. Because the xylocopine tribal crown ages cluster at approximately half the total tree height, any change in the root-node age leads to corresponding changes in tribal crown ages of about half that value. The only fossil apid prior to the Eocene is the meliponine species *Cretotrigona prisca*, from the late Cretaceous [51]. Using molecular analyses, Cardinal and Danforth [2] estimated a crown age for extant Meliponini of approximately 55 Mya and a stem age of nearly 70 Mya. Therefore, whilst the age of *C. prisca* does not rule out the timeframe for basal Apidae divergences estimated by [2], it is also concordant with the older node-age estimates from [11] which suggested a most recent common ancestor between Xylocopinae and the corbiculates at >100 Mya.

The second important issue when interpreting our LTT plots is the existence of a lag-time between an event of massive extinction or a radical change in λ/μ (i.e. speciation/extinction) ratios and a corresponding upturn in LTT curves. Both our MEE and LFM simulations (Figure 3) show sharp discontinuities in lineage accumulation after these simulated events, but in both cases the majority of simulations showed an upturn in lineage accumulation that lagged significantly behind the events themselves. Whilst this may seem counterintuitive, extinction rates associated with a Yule process when lineage numbers are relatively small will mean that some lineages generated shortly after the 65 Mya event would have been lost before they could themselves give rise to new lineages. The effect of this kind of stochastic variation on LTT curves would decrease over time as the number of lineages gradually increases and random birth and death events are averaged over an increasing number of lineages. Consequently, if the K-T or closely associated events played an important role in the cladogenesis of Xylocopinae, the apparent lag time between this event(s) and subsequent upturns in the empirical post-burnin LTT plots (Figure 2) are concordant with our simulation models. Interestingly, the lag time in our empirical LTT plots is shorter than for most of our simulated plots. Our simulations all involved a constant λ/μ beginning at 65 Mya and at ending at the present, whereas diversification after a massive extinction event would have involved ecological radiations into many unoccupied niches, so we could expect initially high speciation rates that gradually decline over time as available niches become successively occupied (e.g. [52], [53]). This would also help explain the bowed curve in our empirical LTT plots, in contrast to the linear plots from our simulations.

The third important issue for interpreting our LTT curves is whether they are better explained by a massive extinction event (MEE) or a ‘Long Fuse’ model (LFM), where early diversification is followed by a period of relatively low diversification rate, after which elevated cladogenesis is restored [43]. We explored a very wide range of MEE and LFM scenarios in simulations by varying speciation (λ) and extinction (μ) absolute and relative values, but these simulations all involved break points of 90 and 65 Mya for 3-phase models, or else an extinction event at 65 Mya. Much more complex models are possible, but we found that the major patterns in our observed LTT plots were captured in these simplified parameter spaces. We found that both MEE and LFM scenarios were able to produce LTT plots that broadly matched the main patterns of our observed data (Figure 3).

We do not suggest that the parameter values in our simulations correspond to the actual speciation and extinction rates over the approximately 100 million year history of the Xylocopinae, and both simulation model types could have been further elaborated by increasing model complexity to arbitrarily increase the fit between simulated and observed data. However, our simulations suggest that our observed data are concordant with both MEE and LFM scenarios, so that discriminating between these possible alternatives will depend on biological arguments. We now explore these.

A long-fuse model would require that a very early capacity for radiation close to the origin of the Xylocopinae was then followed by a period of 20–30 million years where rates of diversification were so low that they did not result in any new clade accumulation within this very long time-span. A long-fuse model would also require that this long period of stasis was then followed by dramatically increased rates of cladogenesis in the Xylocopini, Ceratinini and Allodapini. Importantly, these tribes differ in both their nesting biology and forms of sociality [4], [46] as well as their inferred centers of origin. Historical biogeographic analyses suggest that Xylocopini originated in Asia [31], Allodapini originated in Africa [37] and Ceratinini arose in either Africa or had a joint African/Asian origin [12]. Whilst Manueliini contains only three species, it also has a long stem, but differs from the other tribes in nesting biology and sociality [54], and is restricted to South America. A long-fuse model would therefore require key and independent adaptations in each of the tribes that allowed them to all undergo radiation at similar times, despite a very long period of time where cladogenesis was low or absent. Furthermore, this long period of low or zero diversification would have occurred when eudicot diversification rates were high [49], [50], [55] and would contradict the often suggested coevolution between flowering plants and pollinators in the late Cretaceous [56]. In summary, a long fuse model would require that Xylocopinae experienced almost no diversification over a period of 20–30 million years in the latter part of the Cretaceous, despite eudicots showing rapid diversification over this time, but then experienced sudden resumptions in diversification across multiple continents and in very different tribes. We argue that this scenario, requiring multiple coincident events, is much less likely than a MEE, provided that such an extinction event impacted bee faunas in Africa, Asia and South America.

A massive extinction event at the K-T boundary affecting bees is not unexpected, given evidence for disruption of other plant-insect relationships at that time [21] and evidence that massive extinctions of angiosperms were as widely separated as North America [57] and New Zealand [58], [59]. The global extinction of non-avian dinosaurs also suggests that events surrounding the K-T boundary had massive and geographically widespread effects on terrestrial ecosystems.

The only other study to examine patterns of lineage accumulation in bees over a time period encompassing the K-T event was by Almeida et al. [9] on the bee family Colletidae and they did not find LTT signatures for a K-T extinction event. However, that study recovered a crown age for Colletidae of approximately 71 Mya, with a lower 95% HPD limit of 57 Mya, some 8 My after the K-T event. It is therefore possible that the extant Colletidae are derived from a single lineage that managed to survive the K-T event and which subsequently underwent rapid diversification, giving rise to the eight or more subfamilies [10] now in that clade.

At present it is not possible to determine whether other bee groups might have suffered MEEs corresponding to the K-T event and surrounding period. Cardinal and Danforth's [2] study provides stem ages for a large number of higher bee clades, but exploring whether and MEE or LFM might apply to those taxa requires dense sampling of lineages within each major clade to determine the crown age of extant lineages, and molecular data do not currently exist to allow that. However, this is an issue that deserves attention. We know that changes in pollinator diversity affect diversification in associated plants [55], so an MEE affecting bees is likely to have impacted on subsequent eudicot radiations. Any stochasticity in which bee lineages managed to survive the K-T event therefore has the potential to be a major driver in how subsequent diversity evolved. Given current concerns about looming potential losses in diversity of pollinating insects in general [60], [61], and bees in particular [62]–[64], it is becoming important to understand how pollinators responded to global perturbations in the past and what the future short-term and long-term consequences for plant-pollinator relationships might be.

## Supporting Information

Figure S1 **Maximum credibility tree from our BEAST analysis indicating posterior probability support values for nodes.**
(PDF)

Figure S2 **Maximum credibility tree from our BEAST analysis with purple bars indicating 95% HPDs for node ages.** The root node was fixed at 107 Mya.
(PDF)

Figure S3 **Consensus phylogram from our MrBayes analysis, transformed into a chronogram using penalized likelihood implemented in r8s 1.71.** Node values indicate poster probabilities.
(PDF)

Figure S4 **LTT plots using TreeSim and the same parameters as in** **Figure 3(a)** **, but with extinct lineages also included (200 simulations are graphed).** This figure indicates the likely range in the number of lineages present immediately before the extinction event (represented by the vertical blue line), given the simulation model parameters.
(PDF)

Table S1 **Genbank accession numbers for sequences used in the study.**
(DOC)
