# Supplementary figures and images for: First Evidence for a Massive Extinction Event Affecting Bees Close to the K-T Boundary
Source: PLoS One. 2013 Oct 23;8(10):e76683. doi: 10.1371/journal.pone.0076683 (PMC3806776; doi:10.1371/journal.pone.0076683)

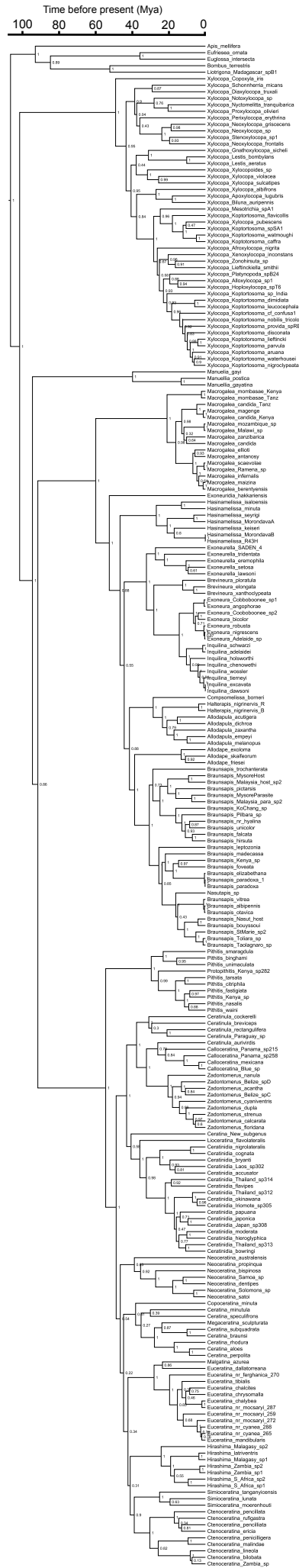

Supplement: Figure S1 — Maximum credibility tree from our BEAST analysis indicating posterior probability support values for nodes. (PDF) [file pone.0076683.s001.pdf]

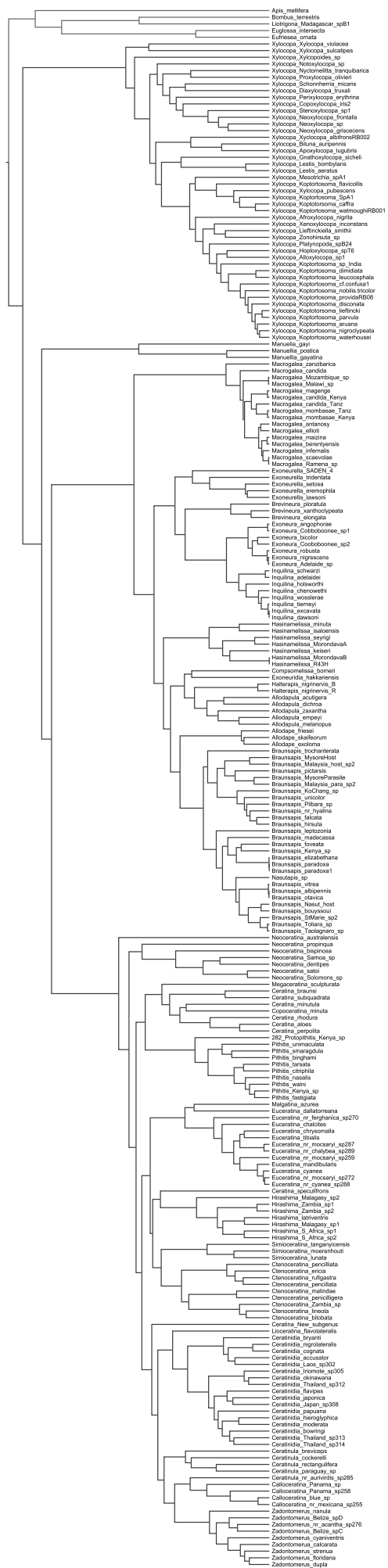

Supplement: Figure S2 — Maximum credibility tree from our BEAST analysis with purple bars indicating 95% HPDs for node ages. The root node was fixed at 107 Mya. (PDF) [file pone.0076683.s002.pdf]

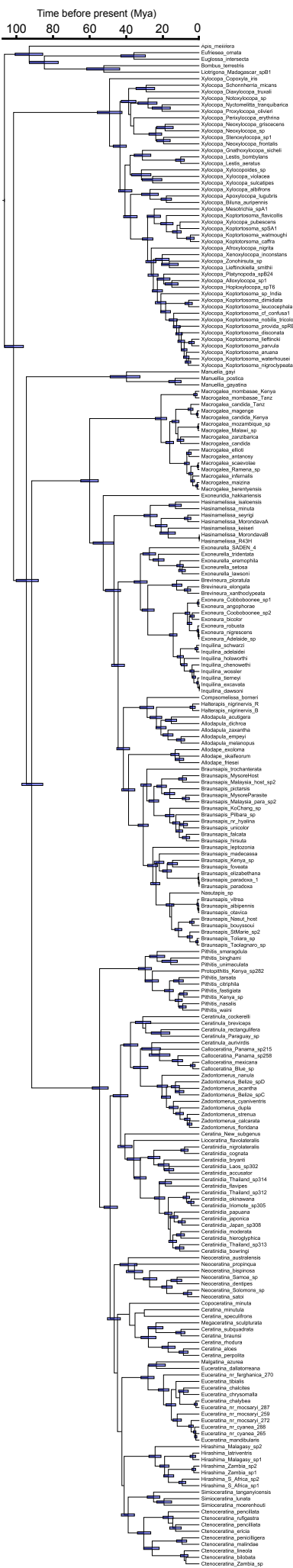

Supplement: Figure S3 — Consensus phylogram from our MrBayes analysis, transformed into a chronogram using penalized likelihood implemented in r8s 1.71. Node values indicate poster probabilities. (PDF) [file pone.0076683.s003.pdf]

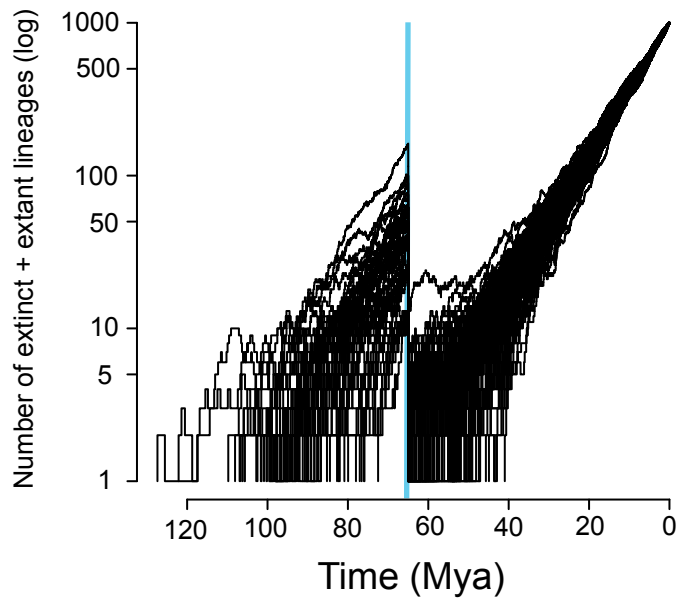

Supplement: Figure S4 — LTT plots using TreeSim and the same parameters as in Figure 3(a) , but with extinct lineages also included (200 simulations are graphed). This figure indicates the likely range in the number of lineages present immediately before the extinction event (represented by the vertical blue line), given the simulation model parameters. (PDF) [file pone.0076683.s004.pdf]
