# Supplementary material for: First Evidence for a Massive Extinction Event Affecting Bees Close to the K-T Boundary
Source: PLoS One. 2013 Oct 23;8(10):e76683. doi: 10.1371/journal.pone.0076683 (PMC3806776; doi:10.1371/journal.pone.0076683)
Supplement: Table S1 — Genbank accession numbers for sequences used in the study. (DOC) [file pone.0076683.s005.doc]

**Table S1.** Genbank accession numbers for sequences used in the study.

| Genus_species | COI | cytb | EF1A-F1 | EF1A-F2 |
| --- | --- | --- | --- | --- |
| *Allodape_exoloma* | KC351806 / KC351787 | KC351768 | N/A | KC351755 |
| *Allodape_friesei* | DQ149656 | DQ149683 | DQ149694 | DQ149701 |
| *Allodape_skaifeorum* | AJ416801 | AJ416829 | N/A | N/A |
| *Allodapula_acutigera* | AY247265 | AY247266 | N/A | N/A |
| *Allodapula_dichroa* | HQ270144 / HQ285963 | HQ285964 | N/A | N/A |
| *Allodapula_empeyi* | DQ149659 | DQ149684 | DQ149695 | DQ149704 |
| *Allodapula_melanopus* | JN564690 | JN564696 | N/A | JN564702 |
| *Allodapula_zaxantha* | HM013827 | HM013828 | N/A | HM013829 |
| *Braunsapis_albipennis* | AJ416804 | AJ416832 | N/A | AJ416777 |
| *Braunsapis_bouyssoui* | AJ416805 | AJ416833 | N/A | NA |
| *Braunsapis_elizabethana* | KC351812 / KC351794 | KC351775 | N/A | N/A |
| *Braunsapis_falcata* | KC351790 | KC351771 | N/A | N/A |
| *Braunsapis_foveata* | EF190098 | EF190105 | N/A | EF190112 |
| *Braunsapis_hirsuta* | KC351810 / KC351792 | KC351773 | N/A | KC351759 |
| *Braunsapis_*Kenya_sp | AJ416809 | AJ416837 | N/A | N/A |
| *Braunsapis_*KoChang_sp | JN564692 | JN564698 | N/A | JN564703 |
| *Braunsapis_leptozonia* | EF190097 | EF190104 | N/A | NA |
| *Braunsapis_madecassa* | DQ160166 | NA | N/A | DQ160174 |
| *Braunsapis_*Malaysia_host_sp2 | JN426778 | JN426790 | N/A | JN426801 |
| *Braunsapis_*Malaysia_para_sp2 | KC351820 / KC351805 | KC351785 | N/A | KC351767 |
| *Braunsapis_*MysoreHost | KC351808 / KC351789 | KC351770 | N/A | KC351757 |
| *Braunsapis_*MysoreParasite | KC351807 / KC351788 | KC351769 | N/A | KC351756 |
| *Braunsapis_*Nasut_host | DQ160164 | DQ160170 | N/A | DQ160177 |
| *Braunsapis_nr_hyalina* | KC351809 / KC351791 | KC351772 | N/A | KC351758 |
| *Braunsapis_otavica* | AJ416807 | AJ416835 | N/A | AJ416780 |
| *Braunsapis_paradoxa* | AJ416806 | AJ416834 | N/A | AJ416779 |
| *Braunsapis_pictarsis* | KC351813 | HQ322267 | N/A | HQ322268 |
| *Braunsapis_*Pilbara_sp | KC351811 / KC351793 | KC351774 | N/A | KC351760 |
| *Braunsapis_*StMarie_sp2 | DQ160168 | DQ160172 | N/A | DQ160176 |
| *Braunsapis_*Taolagnaro_sp | DQ160166 | N/A | N/A | DQ160174 |
| *Braunsapis_*Toliara_sp | DQ160165 | N/A | N/A | DQ160173 |
| *Braunsapis_trochanterata* | JN564693 | JN564699 | N/A | JN564704 |
| *Braunsapis_unicolor* | AF072659 / DQ149658 | AF072666 | AJ416776 | DQ149703 |
| *Braunsapis_vitrea* | AJ416808 / DQ149657 | AJ416836 | AJ416781 | DQ149702 |
| *Brevineura_elongata* | AJ416799 / DQ149673 | AJ416827 | AJ416772 | DQ149718 |
| *Brevineura_ploratula* | AJ416796 / DQ149674 | AJ416824 | N/A | AJ416769 |
| *Brevineura_xanthoclypeata* | AJ416798 / DQ149672 | AJ416826 | AJ416771 | DQ149717 |
| *Compsomelissa_borneri* | AJ416812 | AJ416840 | DQ149719 | N/A |
| *Exoneura_*Adelaide_sp | JN426769 | JN426781 | N/A | JN426793 |
| *Exoneura_angophorae* | AJ416786 / DQ149660 | AJ416814 | AJ416759 | DQ149705 |
| *Exoneura_bicolor* | JN426770 | JN426782 | N/A | JN426794 |
| *Exoneura_*Cobboboonee_sp1 | KC351798 | KC351778 | N/A | KC351762 |
| *Exoneura_*Cooboboonee_sp2 | JN426771 | JN426783 | N/A | JN42679 |
| *Exoneura_nigrescens* | AJ416789 / DQ149662 | AJ416817 | AJ416762 | DQ149707 |
| *Exoneura_robusta* | AJ416787 / DQ149661 | AJ416815 | EJ416760 | DQ149706 |
| *Exoneurella_eremophila* | AF072662 / DQ149667 | AF072669 | DQ149696 | DQ149712 |
| *Exoneurella_lawsoni* | AF072661 / DQ149668 | AF02668 | AJ416765 | DQ149713 |
| *Exoneurella_*SADEN_4 | HQ268578 | HQ268554 | N/A | HQ268554 |
| *Exoneurella_setosa* | AJ416795.2 / DQ149666 | AF072671 | AJ416768 | DQ149711 |
| *Exoneurella_tridentata* | AJ416793 / DQ149665 | AF072670 | AJ416766 | DQ149710 |
| *Exoneuridia_hakkariensis* | DQ149678 | DQ149691 | DQ149698 | DQ149722 |
| *Halterapis_nigrinervis*_B | N/A | AJ416841 | N/A | N/A |
| *Halterapis_nigrinervis*_R | JN426780 | JN426792 | N/A | JN426804 |
| *Hasinamelissa_isaloensis* | EU254247 | EU254248 | N/A | N/A |
| *Hasinamelissa_keiseri* | EU814530 | EU814523 | N/A | N/A |
| *Hasinamelissa_minuta* | DQ149676 | DQ149689 | N/A | DQ149720 |
| *Hasinamelissa_*MorondavaA | EU814531 | EU814524 | N/A | EU814525 |
| *Hasinamelissa_*MorondavaB | EU814532 | EU814526 | N/A | EU814527 |
| *Hasinamelissa_*R43H | EU814533 | EU814528 | N/A | EU814529 |
| *Hasinamelissa_seyrigi* | DQ149677 | DQ149690 | N/A | DQ149721 |
| *Inquilina_adelaidei* | KC351800 | KC351780 | N/A | KC351763 |
| *Inquilina_chenowethi* | JN426773 | JN426785 | N/A | JN426797 |
| *Inquilina_dawsoni* | AJ416790 | AJ416818 | AJ416763 | N/A |
| *Inquilina_excavata* | DQ149669 | DQ149697 | DQ149714 | N/A |
| *Inquilina_holsworthi* | KC351816 / KC351801 | KC351781 | N/A | N/A |
| *Inquilina_schwarzi* | DQ149670 | U56094 | AJ416764 | DQ149715 |
| *Inquilina_tierneyi* | JN426772 | JN426784 | N/A | JN426796 |
| *Inquilina_wossler* | JN426774 | JN426786 | N/A | JN426798 |
| *Macrogalea_antanosy* | DQ149680 | DQ149612 | N/A | DQ149724 |
| *Macrogalea_berentyensis* | EF103591 | EF190102 | N/A | EF103601 |
| *Macrogalea_candida* | AJ416810 | AJ416838 | N/A | AJ416783 |
| *Macrogalea_candida_*Kenya | KC351817 /KC351802 | KC351782 | N/A | KC351764 |
| *Macrogalea_candida_*Tanz | JN426775 | JN426787 | N/A | JN426799 |
| *Macrogalea_ellioti* | EF103589 / EF103590 | EF103595 | N/A | EF103600 |
| *Macrogalea_infernalis* | EF103592 | EF103597 | N/A | EF103602 |
| *Macrogalea_magenge* | AY245175 | AY245176 | N/A | AY245174 |
| *Macrogalea_maizina* | EF190094 | EF190101 | N/A | EF190108 |
| *Macrogalea_*Malawi_sp | EF190092 | EF190099 | N/A | N/A |
| *Macrogalea_mombasae_*Kenya | KC351819 / KC351804 | KC351784 | N/A | KC351766 |
| *Macrogalea_mombasae_*Tanz | KC351818 / KC351803 | KC351783 | N/A | KC351765 |
| *Macrogalea_*mozambique_sp | JN426776 | JN426788 | N/A | JN426800 |
| *Macrogalea_*Ramena_sp | EF103593 | EF103598 | N/A | EF103603 |
| *Macrogalea_scaevolae* | EF190096 | EF190103 | N/A | EF190110 |
| *Macrogalea_zanzibarica* | AJ416811 / DQ149679 | AY625455 | DQ149699 | DQ149723 |
| *Nasutapis_*sp | DQ160163 | DQ160169 | N/A | DQ160178 |
| *Ceratina_Calloceratina_*Blue_sp | GU321504 | N/A | N/A | GU321639 |
| *Ceratina_Calloceratina_mexicana* | JX968036 | JX968060 | N/A | JX968084 |
| *Ceratina_Calloceratina_*Panama_sp215 | JX968035 | JX968061 | N/A | JX968083 |
| *Ceratina_Calloceratina_*Panama_sp258 | GU321508 | GU321574 | N/A | GU321643 |
| *Ceratina_Ceratina_aloes* | GU321535 | GU321600 | N/A | GU321670 |
| *Ceratina_Ceratina_braunsi* | GU321532 | GU321597 | JQ230039 | N/A |
| *Ceratina_Ceratina_minutula* | GU321671 | GU321601 | N/A | GU321643 |
| *Ceratina_* New_subgenus | N/A | GU321586 | JQ230041 | GU321656 |
| *Ceratina_Ceratina_perpolita* | GU321538 | N/A | N/A | GU321673 |
| *Ceratina_Ceratina_rhodura* | GU321537 | GU321602 | JQ230040 | GU321672 |
| *Ceratina_Ceratina_speculifrons* | GU321533 | GU321598 | N/A | GU321668 |
| *Ceratina_Ceratina_subquadrata* | GU321534 | GU321599 | N/A | GU321669 |
| *Ceratina_Ceratinidia_accusator* | GU321475 | GU321547 | N/A | GU321610 |
| *Ceratina_Ceratinidia_bowringi* | GU321476 | GU321548 | N/A | GU321611 |
| *Ceratina_Ceratinidia_bryanti* | GU321477 | GU321549 | N/A | GU321612 |
| *Ceratina_Ceratinidia_cognata* | GU321473 | GU321545 | N/A | GU321608 |
| *Ceratina_Ceratinidia_flavipes* | JQ230011 | JQ230006 | JQ230022 | N/A |
| *Ceratina_Ceratinidia_hieroglyphica* | GU321479 | GU321551 | N/A | GU321614 |
| *Ceratina_Ceratinidia_*Iriomote_sp305 | JX968039 | JX968077 | N/A | JX968086 |
| *Ceratina_Ceratinidia_*Japan_sp308 | JX968040 | JX968079 | N/A | JX968088 |
| *Ceratina_Ceratinidia_japonica* | GU321470 | GU321542 | JQ230020 | GU321605 |
| *Ceratina_Ceratinidia_*Laos_sp302 | JX968037 | JX968076 | N/A | JX968085 |
| *Ceratina_Ceratinidia_moderata* | GU321472 | GU321544 | N/A | GU321607 |
| *Ceratina_Ceratinidia_nigrolateralis* | GU321471 | GU321543 | N/A | GU321606 |
| *Ceratina_Ceratinidia_okinawana* | GU321478 | GU321550 | JQ230002 | GU321613 |
| *Ceratina_Ceratinidia_papuana* | GU321474 | GU321546 | N/A | GU321609 |
| *Ceratina_Ceratinidia_*Thailand_sp312 | JX968041 | JX968080 | N/A | JX968089 |
| *Ceratina_Ceratinidia_*Thailand_sp313 | JX968042 | JX968081 | N/A | JX968090 |
| *Ceratina_Ceratinidia_*Thailand_sp314 | JX968044 | JX968082 | N/A | JX968091 |
| *Ceratina_Ceratinula_aurivirdis* | JX968038 | N/A | N/A | JX968092 |
| *Ceratina_Ceratinula_breviceps* | GU321507 | GU321573 | N/A | GU321642 |
| *Ceratina_Ceratinula_cockerelli* | GU321641 | N/A | N/A | GU321641 |
| *Ceratina_Ceratinula_*Paraguay_sp | GU321500 | GU321568 | N/A | GU321635 |
| *Ceratina_Ceratinula_rectangulifera* | JQ230013 | JQ230008 | N/A | JQ230057 |
| *Ceratina_Copoceratina_minuta* | GU321531 | N/A | N/A | GU321667 |
| *Ceratina_Ctenoceratina_bilobata* | GU321491 | GU321561 | N/A | GU321626 |
| *Ceratina_Ctenoceratina_ericia* | GU321489 | GU321559 | JQ230027 | GU321624 |
| *Ceratina_Ctenoceratina_lineola* | GU321495 | GU321563 | N/A | GU321630 |
| *Ceratina_Ctenoceratina_malindae* | GU321496 | GU321564 | JQ230026 | GU321631 |
| *Ceratina_Ctenoceratina_pencillata* | GU321495 | GU321563 | N/A | GU321630 |
| *Ceratina_Ctenoceratina_pencillata* | GU321497 | GU321565 | N/A | GU321632 |
| *Ceratina_Ctenoceratina_penicilligera* | GU321494 | N/A | N/A | GU321629 |
| *Ceratina_Ctenoceratina_rufigastra* | GU321493 | N/A | N/A | GU321628 |
| *Ceratina_Ctenoceratina_*Zambia_sp | GU321490 | GU321560 | N/A | GU321625 |
| *Ceratina_Euceratina_chalcites* | GU321485 | N/A | JQ230024 | GU321620 |
| *Ceratina_Euceratina_chalybea* | JX968047 | JX968073 | N/A | JX968095 |
| *Ceratina_Euceratina_chrysomalla* | GU321485 | N/A | N/A | GU321620 |
| *Ceratina_Euceratina_dallatorreana* | JX968045 | JX968064 | N/A | JX968093 |
| *Ceratina_Euceratina_mandibularis* | GU321482 | GU321554 | N/A | GU321617 |
| *Ceratina_Euceratina_*nr_cyanea_265 | JX968051 | JX968065 | N/A | JX968099 |
| *Ceratina_Euceratina_*nr_cyanea_288 | JX968057 | JX968072 | N/A | JX968101 |
| *Ceratina_Euceratina_*nr_ferghanica_270 | JX968046 | JX968067 | N/A | JX968094 |
| *Ceratina_Euceratina_*nr_mocsaryi_259 | JX968048 | JX968062 | N/A | JX968096 |
| *Ceratina_Euceratina_*nr_mocsaryi_272 | JX968050 | JX968068 | N/A | JX968098 |
| *Ceratina_Euceratina_*nr_mocsaryi_287 | JX968056 | JX968071 | N/A | JX968100 |
| *Ceratina_Euceratina_tibialis* | GU321484 | GU321556 | N/A | GU321619 |
| *Ceratina_Hirashima_latriventris* | GU321514 | GU321579 | JQ230032 | GU321649 |
| *Ceratina_Hirashima_*Malagasy_sp1 | GU321509 | N/A | N/A | GU321644 |
| *Ceratina_Hirashima_*Malagasy_sp2 | GU321510 | GU321575 | N/A | GU321645 |
| *Ceratina_Hirashima_*S_Africa_sp1 | GU321483 | GU321555 | JQ230033 | GU321618 |
| *Ceratina_Hirashima_*S_Africa_sp2 | GU321511 | GU321576 | JQ230034 | GU321646 |
| *Ceratina_Hirashima_*Zambia_sp1 | GU321515 | GU321580 | N/A | GU321650 |
| *Ceratina_Hirashima_*Zambia_sp2 | GU321512 | GU321577 | N/A | GU321647 |
| *Ceratina_Lioceratina_flavolateralis* | GU321513 | GU321578 | N/A | GU321648 |
| *Ceratina_Malgatina_azurea* | GU321481 | GU321553 | JQ230023 | GU321616 |
| *Ceratina_Megaceratina_sculpturata* | GU321498 | GU321566 | N/A | GU321633 |
| *Ceratina_Neoceratina_australensis* | GU321616 | GU321553 | JQ230015 | GU321616 |
| *Ceratina_Neoceratina_bispinosa* | GU321521 | GU321587 | JQ230018 | GU321657 |
| *Ceratina_Neoceratina_dentipes* | GU321516 | GU321581 | JQ230016 | GU321651 |
| *Ceratina_Neoceratina_propinqua* | GU321520 | GU321581 | JQ230017 | GU321655 |
| *Ceratina_Neoceratina_Samoa_sp* | JQ230010 | N/A | JQ230014 | JQ230055 |
| *Ceratina_Neoceratina_satoi* | GU321518 | GU321583 | JQ230019 | GU321653 |
| *Ceratina_Neoceratina_*Solomons_sp | GU321517 | GU321582 | N/A | GU321652 |
| *Ceratina_Pithitis_binghami* | GU321526 | GU321592 | JQ230037 | GU321662 |
| *Ceratina_Pithitis_citriphila* | GU321525 | GU321591 | N/A | GU321661 |
| *Ceratina_Pithitis_fastigiata* | GU321539 | GU321603 | N/A | GU321674 |
| *Ceratina_Pithitis_*Kenya_sp | GU321527 | GU321593 | JQ230038 | GU321663 |
| *Ceratina_Pithitis_nasalis* | GU321528 | GU321594 | N/A | GU321664 |
| *Ceratina_Pithitis_smaragdula* | GU321523 | GU321589 | JQ230035 | GU321659 |
| *Ceratina_Pithitis_tarsata* | GU321530 | GU321596 | JQ230036 | GU321666 |
| *Ceratina_Pithitis_unimaculata* | GU321519 | GU321584 | N/A | GU321654 |
| *Ceratina_Pithitis_waini* | GU321529 | GU321595 | N/A | GU321665 |
| *Ceratina_Protopithitis_*Kenya_sp282 | JX968053 | JX968070 | N/A | JX968103 |
| *Ceratina_Simioceratina_lunata* | GU321522 | GU321588 | N/A | GU321658 |
| *Ceratina_Simioceratina_moerenhouti* | GU321486 | GU321557 | JQ230025 | GU321621 |
| *Ceratina_Simioceratina_tanganyicensis* | GU321524 | GU321590 | N/A | GU321660 |
| *Ceratina_Zadontomerua_calcarata* | GU321499 | GU321567 | JQ230028 | GU321634 |
| *Ceratina_Zadontomerus_acantha* | JX968059 | JX968069 | N/A | JX968107 |
| *Ceratina_Zadontomerus_*Belize_spC | JX968054 | N/A | N/A | JX968104 |
| *Ceratina_Zadontomerus_*Belize_spD | JX968058 | JX968075 | N/A | JX968106 |
| *Ceratina_Zadontomerus_cyaniventris* | GU321503 | GU321571 | N/A | GU321638 |
| *Ceratina_Zadontomerus_dupla* | GU321488 | N/A | N/A | GU321623 |
| *Ceratina_Zadontomerus_floridana* | GU321487 | GU321558 | JQ230029 | GU321622 |
| *Ceratina_Zadontomerus_nanula* | JQ230012 | JQ230007 | JQ230031 | JQ230056 |
| *Ceratina_Zadontomerus_strenua* | GU321505 | GU321572 | JQ230030 | GU321640 |
| *Manuelia_gayi* | HM461879 | HM461882 | HM461885 | GU321604 |
| *Manuellia_gayatina* | HM461878 | HM461881 | HM461884 | N/A |
| *Manuellia_postica* | HM461880 | HM461883 | HM461886 | N/A |
| *Xylocopa_Afroxylocopa_nigrita* | AY005238 | AY005265 | AY005292 | JQ230045 |
| *Xylocopa_Alloxylocopa*_sp1 | KC136021 | AY005270 | EU445976 | N/A |
| *Xylocopa_Apoxylocopa_lugubris* | EU180090 | EU180106 | EU180118 | JQ230047 |
| *Xylocopa_Biluna_auripennis* | AY005225 | AY005252 | AY005279 | JQ230046 |
| *Xylocopa_Copoxyla_iris* | AY005232 | AY005259 | AY005286 | N/A |
| *Xylocopa_Diaxylocopa_truxali* | AY005247 | AY005274 | AY005301 | N/A |
| *Xylocopa_Gnathoxylocopa_sicheli* | AY005230 | AY005257 | AY005284 | JQ230048 |
| *Xylocopa_Hoploxylocopa_*spT6 | AY005242 | AY005269 | AY005296 | N/A |
| *Xylocopa_Koptortosoma_aruana* | AY005234 | AY005261 | AY005288 | N/A |
| *Xylocopa_Koptortosoma_cf_confusa1* | EU861304 | N/A | EU445986 | N/A |
| *Xylocopa_Koptortosoma_dimidiata* | EU180079 | EU180098 | EU180109 | N/A |
| *Xylocopa_Koptortosoma_disconata* | EU180075 | EU180094 | N/A | N/A |
| *Xylocopa_Koptortosoma_flavicollis* | EU180082 | EU180101 | EU180111 | N/A |
| *Xylocopa_Koptortosoma_leucocephala* | EU180080 | EU180099 | N/A | N/A |
| *Xylocopa_Koptortosoma_nigroclypeata* | EU180074 | EU180093 | N/A | N/A |
| *Xylocopa_Koptortosoma_nobilis_tricolor* | EU180078 | EU180097 | EU180108 | N/A |
| *Xylocopa_Koptortosoma_parvula* | EU180073 | EU180092 | N/A | N/A |
| *Xylocopa_Koptortosoma_provida*_spRB08 | EU180088 | EU180105 | EU180116 | N/A |
| *Xylocopa_Koptortosoma_*sp_India | EU180081 | EU180100 | EU180110 | N/A |
| *Xylocopa_Koptortosoma_*spSA1 | AY005237 | AY005264 | AY005291 | N/A |
| *Xylocopa_Koptortosoma_waterhousei* | EU180076 | EU180095 | N/A | N/A |
| *Xylocopa_Koptortosoma_watmoughi* | EU180087 | N/A | EU180115 | N/A |
| *Xylocopa_Koptotorsoma_caffra* | EU180083 | EU180102 | EU180112 | JQ230050 |
| *Xylocopa_Koptotorsoma_lieftincki* | AY005235 | AY005262 | AY005289 | JQ230051 |
| *Xylocopa_Lestis_aeratus* | EU180091 | EU180107 | EU180119 | JQ230053 |
| *Xylocopa_Lestis_bombylans* | AY005227 | AY005254 | AY005281 | JQ230052 |
| *Xylocopa_Lieftinckiella_smithii* | EU180085 | EU180104 | EU180114 | N/A |
| *Xylocopa_Mesotrichia_*spA1 | AY005239 | AY005266 | AY005293 | N/A |
| *Xylocopa_Neoxylocopa_frontalis* | AY005248 | AY005275 | AY005302 | JQ230049 |
| *Xylocopa_Neoxylocopa_griscecens* | JQ230009 | N/A | N/A | JQ230054 |
| *Xylocopa_Neoxylocopa*_sp | AY005245 | AY005272 | AY005299 | N/A |
| *Xylocopa_Notoxylocopa_*sp | AY005228 | AY005255 | AY005282 | N/A |
| *Xylocopa_Nyctomelitta_tranquibarica* | AY005224 | AY005251 | AY005278 | JQ230044 |
| *Xylocopa_Perixylocopa_erythrina* | AY005229 | AY005256 | AY005283 | N/A |
| *Xylocopa_Platynopoda_*spB24 | AY005241 | AY005268 | AY005295 | N/A |
| *Xylocopa_Proxylocopa_olivieri* | AY005240 | AY005267 | AY005294 | N/A |
| *Xylocopa_Schonnherria_micans* | AY005223 | AY005250 | AY005277 | N/A |
| *Xylocopa_Stenoxylocopa_*sp1 | AY005244 | AY005271 | AY005298 | N/A |
| *Xylocopa_Xenoxylocopa_inconstans* | EU180084 | EU180103 | EU180113 | N/A |
| *Xylocopa_Xylocopa_albifrons* | EU180089 | N/A | EU180117 | N/A |
| *Xylocopa_Xylocopa_pubescens* | AY005236 | AY005263 | AY005290 | JQ230043 |
| *Xylocopa_Xylocopa_sulcatipes* | AY005233 | AY005260 | AY005287 | N/A |
| *Xylocopa_Xylocopa_violacea* | AY005226 | AY005253 | AY005280 | JQ230042 |
| *Xylocopa_Xylocopoides_*sp | AY005231 | AF181618 | AY005285 | AY208290 |
| *Xylocopa_Zonohirsuta_*sp | EU180086 | N/A | EU445989 | N/A |

***N/A indicates no sequence available**
